# Dominant Mutations in *GRM1* Cause Spinocerebellar Ataxia Type 44

**DOI:** 10.1016/j.ajhg.2017.08.005

**Published:** 2017-09-07

**Authors:** Lauren M. Watson, Elizabeth Bamber, Ricardo Parolin Schnekenberg, Jonathan Williams, Conceição Bettencourt, Jennifer Lickiss, Katherine Fawcett, Samuel Clokie, Yvonne Wallis, Penny Clouston, David Sims, Henry Houlden, Esther B.E. Becker, Andrea H. Németh

**Affiliations:** 1 Department of Physiology, Anatomy and Genetics, University of Oxford, Oxford OX1 3PT, UK; 2 Nuffield Department of Clinical Neurosciences, University of Oxford, 6th Floor West Wing, John Radcliffe Hospital, Oxford OX3 9DU, UK; 3 Oxford Medical Genetics Laboratories, Churchill Hospital, Oxford OX3 7LE, UK; 4 Department of Molecular Neuroscience, Institute of Neurology, University College London, London WC1N 3BG, UK; 5 Department of Clinical and Experimental Epilepsy, Institute of Neurology, University College London, London WC1N 3BG, UK; 6 West Midlands Regional Genetics Laboratory, Birmingham Women’s and Children’s NHS Foundation Trust, Birmingham B15 2TG, UK; 7 MRC Computational Genomics Analysis and Training Programme, MRC Weatherall Institute of Molecular Medicine, University of Oxford, John Radcliffe Hospital, Oxford OX3 9DS, UK; 8 Oxford Centre for Genomic Medicine, Oxford University Hospitals NHS Trust, Oxford OX3 7HE, UK

**Keywords:** mGluR1, glutamate, calcium, cerebellum, ataxia, atrophy, intellectual disability, Purkinje cells, Homer, nitazoxanide

## Abstract

The metabotropic glutamate receptor 1 (mGluR1) is abundantly expressed in the mammalian central nervous system, where it regulates intracellular calcium homeostasis in response to excitatory signaling. Here, we describe heterozygous dominant mutations in *GRM1*, which encodes mGluR1, that are associated with distinct disease phenotypes: gain-of-function missense mutations, linked in two different families to adult-onset cerebellar ataxia, and a *de novo* truncation mutation resulting in a dominant-negative effect that is associated with juvenile-onset ataxia and intellectual disability. Crucially, the gain-of-function mutations could be pharmacologically modulated *in vitro* using an existing FDA-approved drug, Nitazoxanide, suggesting a possible avenue for treatment, which is currently unavailable for ataxias.

## Main Text

Glutamate is the most abundant excitatory neurotransmitter in the mammalian brain.^1^ Postsynaptic glutamate signaling is mediated by two classes of receptors: ionotropic glutamate receptors (iGluRs), which mediate rapid synaptic transmission; and metabotropic glutamate receptors (mGluRs), which are coupled to G proteins and produce a more complex postsynaptic response consisting of both internal calcium release and a slow excitatory postsynaptic potential. mGluR1, encoded by *GRM1* (MIM: 604473), is one of the most abundant mGluRs in the mammalian central nervous system and is present at particularly high levels in Purkinje cells, the primary neurons of the cerebellar cortex. Multiple lines of evidence have implicated mGluR1 as a central player in diseases involving glutamatergic dysfunction and abnormal synaptic plasticity.^2^, ^3^ Nevertheless, disease-causing mutations within *GRM1* itself appear remarkably rare.^4^ The only *GRM1* mutations identified to date have been found either to cause an autosomal-recessive spinocerebellar ataxia (SCAR13 [MIM: 614831]) in a small Roma cohort with a known founder effect^5^ or to associate with autosomal-recessive intellectual disability in a single consanguineous Iranian family.^6^

**Figure 1:**
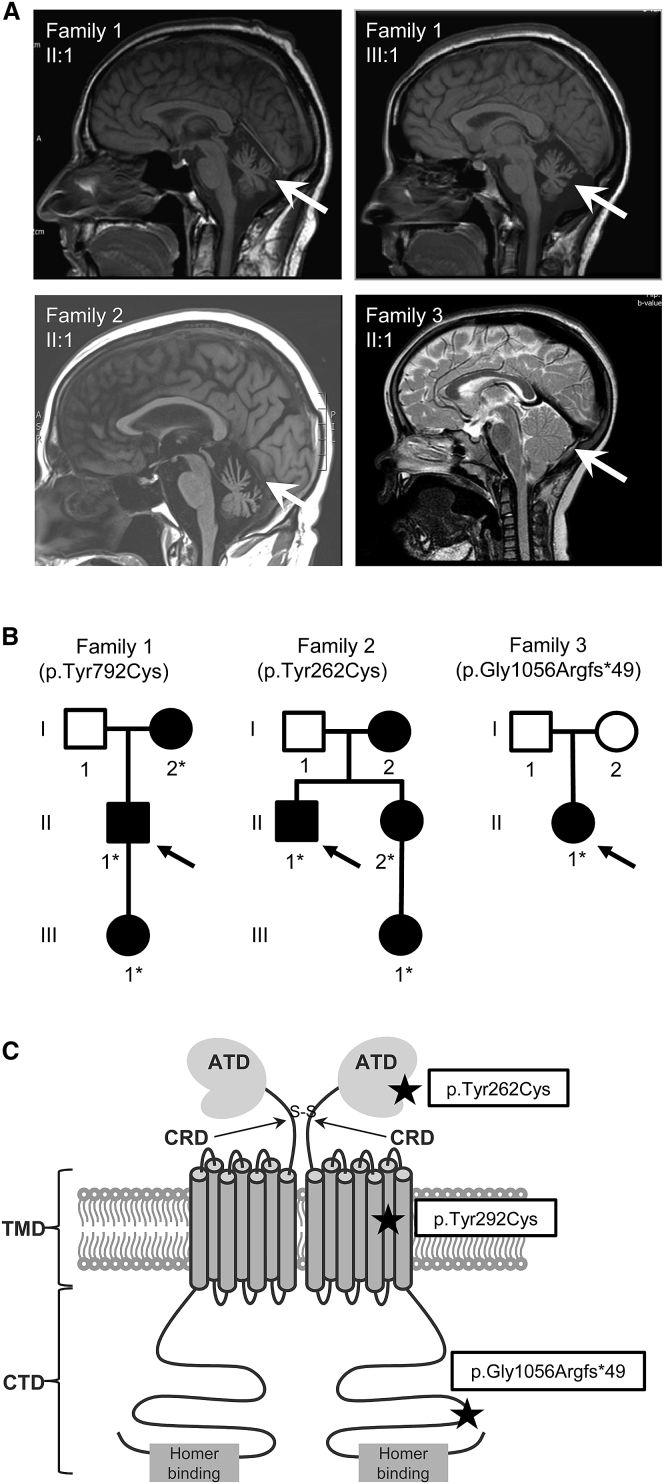
Dominant Mutations in *GRM1* Result in a Cerebellar Phenotype (A) MRI brain imaging of case subjects. Top left: family 1, affected son (II:1); top right: family 1, affected granddaughter (III:1), both showing cerebellar atrophy. Bottom left: family 2, affected brother (II:1) showing cerebellar atrophy; bottom right: family 3, affected daughter (II:1), showing normal imaging. The cerebellum is indicated in each case by an arrow. (B) Pedigrees of affected families. Squares denote male family members, circles female family members, and black symbols affected family members. Probands are indicated in each case by an arrow. The following individuals were sequenced: family 1 I:2, II:1, and III:1; family 2 II:1, II:2, and III:1; family 3 I:1, I:2, and II:1. Asterisks (^∗^) indicate the presence of the mutation. (C) Schematic representation of the positions of the dominant mutations within mGluR1. At the N terminus, the amino-terminal domain (ATD) is followed by the cysteine-rich domain (CRD), seven transmembrane domains (TMD), and the intracellular C-terminal domain (CTD). Cysteine residues, which function in dimerization, are indicated by S. *GRM1* mutations are indicated by black stars. The p.Tyr262Cys variant is located in the extracellular ligand-binding region, p.Tyr792Cys within transmembrane helix 6, and p.Gly1056Argfs^∗^49 in the C-terminal domain of the receptor. Figure adapted from Willard and Koochekpour.^42^

Here, we report heterozygous dominant mutations in *GRM1* associated with two distinct phenotypes. Missense mutations in *GRM1* were identified in two different families with an adult-onset degenerative disorder primarily causing cerebellar ataxia with some cortical involvement causing spasticity: c.2375A>G (p.Tyr792Cys) in family 1 and c.785A>G (p.Tyr262Cys) in family 2 (Figures 1A and 1B). The clinical presentation in families 1 and 2 is of a slowly progressive cerebellar ataxia with onset between 20 and 50 years (see Supplemental Note). There was no evidence of cognitive impairment, but in family 1, individual III:1 has evidence of corticospinal tract involvement with a narrow stiff gait and brisk reflexes. Brain MRI in members of both families revealed cerebellar atrophy, with mild flattening of the pons in family 1. Genetic testing for spinocerebellar ataxias (SCAs) 1, 2, 3, 6, 7, and 17 (MIM: 164400, 183090, 109150, 183086, 164500, and 607136) did not detect any mutations. We also identified a heterozygous base pair duplication in *GRM1* in another individual (c.3165dup [p.Gly1056Argfs^∗^49]). In family 3 the parents are unaffected, but the child has intellectual disability and cerebellar ataxia without apparent cerebellar atrophy, and normal brain imaging (Figure 1A).

Consent for participation in the study was obtained according to the Declaration of Helsinki (WMA, 1997) and approved by the Central Oxford Research Ethics Committee and the Research and Development Department of the Oxford Radcliffe Hospitals NHS Trust (approval number C03.052), Oxford. Work at University College London Hospitals was conducted under UCLH Project ID Number: 08/0512/26. All participating individuals or their parents provided written consent for the study.

Variants of interest in *GRM1* were identified by means of whole-exome (families 1 and 2) or targeted (family 3) sequencing, with results verified by Sanger sequencing. In the case of family 1, Covaris shearing of DNA was followed by library preparation using the Agilent SureSelect Exome V5 probe kit and SureSelectXT target enrichment chemistry. The prepared libraries were sequenced by 2× 100-bp paired end sequencing on a HiSeq2500 in rapid run mode. A minimum of 98.37% of the on-target regions were covered to a depth of at least 20×. The exome data were processed using an in-house bioinformatic pipeline as previously described.^7^

For family 2, whole-exome sequencing (WES) was performed in the three family members. The TruSeq Exome Enrichment (62 Mb) or the Nextera Rapid Capture Exome (37 Mb) Enrichment kits (Illumina) were used according to the manufacturer instructions. Libraries were sequenced using an Illumina HiSeq2500 using a 100-bp paired-end reads protocol. In the proband, a minimum of 94.66% of the on-target regions were covered to a depth of at least 10×. Sequence alignment to the human reference genome (UCSC hg19) and variants call and annotation were performed using an in-house pipeline as described elsewhere.^8^ The raw list of single-nucleotide variants (SNVs) and indels was then filtered. Only exonic and donor/acceptor splicing variants were considered. Priority was given to rare variants (<1% in public databases, including 1000 Genomes project, NHLBI Exome Variant Server, Complete Genomics 69, and Exome Aggregation Consortium [ExAC v0.2] with a GERP++ score above 2). Synonymous variants were not considered nor were variants present in our in-house exome database in phenotypes other than ataxia.

For family 3, targeted sequencing of 92 ataxia-associated genes (see Table S1) was performed in the proband using a custom design Haloplex enrichment kit (Agilent Technologies) on the Ilumina MiSeq platform. Data analysis was performed using an in-house pipeline. Identified variants were filtered against in-house lists of known sequencing artifacts and polymorphisms and of variants found in the Exome Variant Server dataset at a minor allele frequency of 1% or greater. Horizontal coverage of the target genes at a read-depth of 30× is included in Table S1. The variant was confirmed as having arisen *de novo* by Sanger sequencing of parental DNA, and familial relationships were confirmed using the AmpFLSTR Identifiler Plus PCR amplification kit (ThermoFisher Scientific).

**Figure 2:**
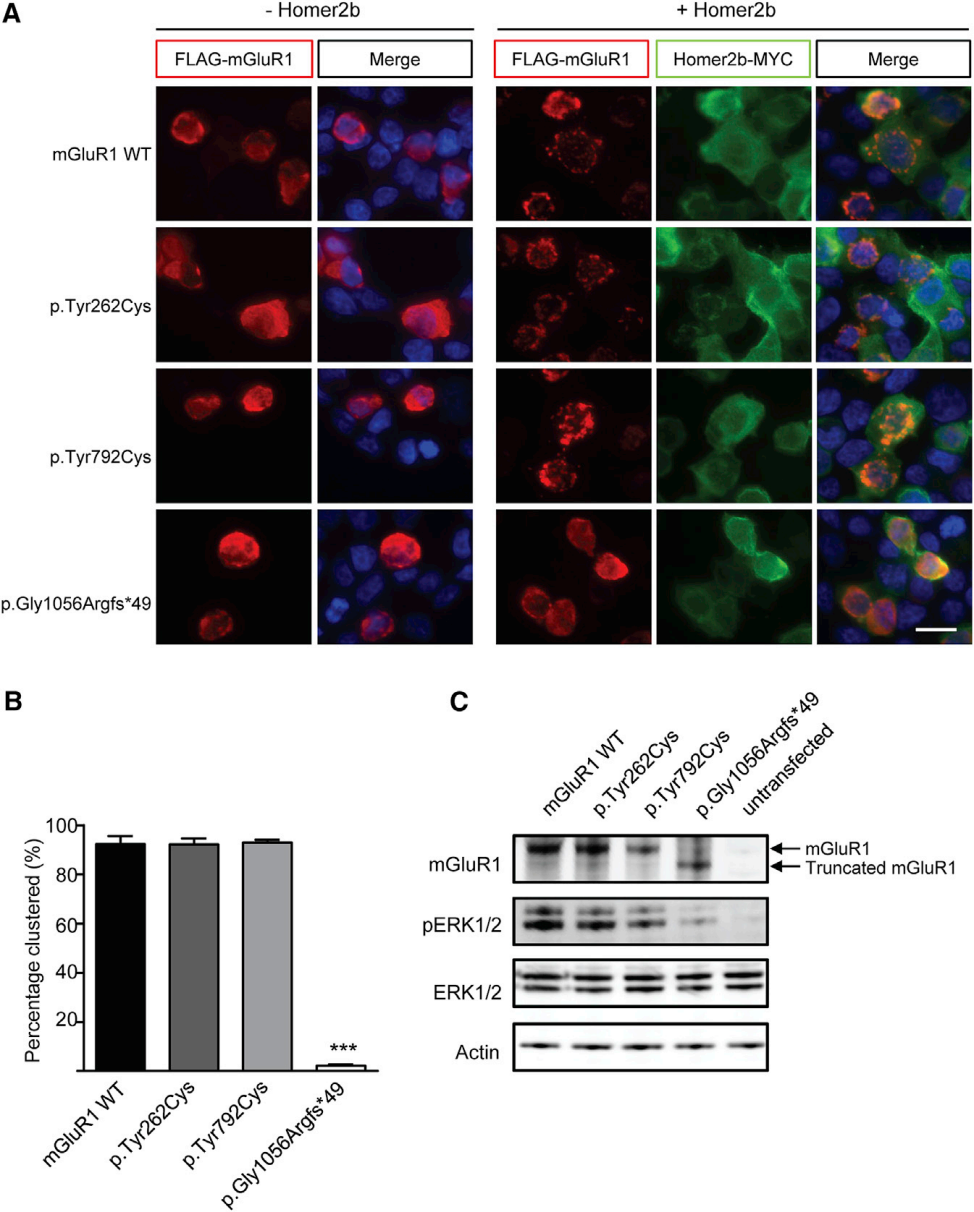
Deletion of the C-Terminal Domain of mGluR1 Affects Binding to Homer2b *GRM1* expression constructs were generated using GRM1-Tango (Addgene plasmid 66387),^15^ into which a stop codon was inserted to prevent readthrough into the Tango element. The three dominant mutations were introduced by site-directed mutagenesis, and results were verified by Sanger sequencing. Constructs were transfected into HEK293FT cells (Invitrogen), using Lipofectamine 3000 (Thermo Fisher Scientific). 24 hr after transfection, cells were subjected to immunostaining using the following primary antibodies: mouse anti-FLAG (1:500; Sigma-Aldrich), rabbit anti-MYC (1:500; Abcam), and goat anti-mGluR1 (1:500; Santa-Cruz). Secondary antibodies: goat anti-mouse Alexa594 or Alexa488, goat anti-rabbit Alexa488, and donkey anti-goat Alexa594 (all 1:1,000; Life Technologies). Nuclei were stained with DAPI. (A) Cells transfected with FLAG-tagged mGluR1 only (left) show diffuse localization of wild-type (WT) and mutant mGluR1 (red). Co-transfection with MYC-tagged Homer2b results in clustering of WT mGluR1 and the p.Tyr262Cys and p.Tyr792Cys mutants but not the p.Gly1056Argfs^∗^49 deletion mutant. Scale bar: 20 μm. (B) Quantitative analysis of mGluR1-Homer2b clustering. For each biological replicate, 100 cells were counted. Bars show the mean of three biological replicates ± SEM. ^∗∗∗^p < 0.001 (one-way ANOVA, followed by Bonferroni’s multiple comparison test). (C) Western blot analysis of mGluR1 and components of its downstream signaling cascade. Protein extracts were prepared from cultured cells 24 hr after transfection, in ice-cold RIPA buffer (Thermo Fisher) containing 1× cOmplete, Mini, EDTA-free Protease Inhibitor Cocktail (Roche), 1× PhosSTOP (Roche), and 1 mM dithiothreitol (DTT), and analyzed by standard SDS-polyacrylamide gel electrophoresis and immunoblotting. Primary antibodies: rabbit anti-mGluR1 (1:200; Alomone Labs), rabbit anti-p44/42 MAPK (Erk1/2) and rabbit anti-phospho-p44/42 MAPK (Erk1/2) (both 1:1,000; Cell Signaling Technologies), and mouse anti-actin (1:1,000; Abcam). HRP-conjugated secondary antibodies: donkey anti-rabbit and sheep anti-mouse (both 1:10,000; GE Healthcare). A decrease in phosphorylation of Extracellular Signal-Related Kinase 1/2 (ERK1/2) was observed in cells transfected with the mGluR1 p.Gly1056Argfs^∗^49 mutant, indicating disruption of mGluR1 downstream signaling events.

Both the heterozygous c.2375A>G (p.Tyr792Cys) (family 1) and c.785A>G (p.Tyr262Cys) (family 2) missense variants were predicted to be pathogenic by standard bioinformatics pathogenicity programs (Table 1). The heterozygous c.3165dup (p.Gly1056Argfs^∗^49) variant identified in the proband of family 3 occurs in the final *GRM1* exon, and as such is not predicted to be subject to nonsense-mediated decay (NMD) but rather to result in the production of a truncated protein. The presence of protein levels for all three variants was confirmed following transient overexpression in HEK293FT cells (Figures 2A and 2C), although further confirmation of p.Gly1056Argfs^∗^49 mGluR1 levels in primary cells would be necessary to conclusively rule out the possibility of NMD.

**Table 1:** Summary of *GRM1* Variants Identified by Sequencing in Affected Families

	**Family 1**	**Family 2**	**Family 3**
Genome reference	GRCh37:g.146720550A>G	GRCh37:g.146480568A>G	GRCh37:g.146755512dup
Transcript	NM_001278064.1	NM_001278064.1	NM_001278064.1
Nucleotide	c.2375A>G	c.785A>G	c.3165dup
Protein	p.Tyr792Cys	p.Tyr262Cys	p.Gly1056Argfs^∗^49
PhyloP	5.05 [-14.1;6.4]	4.56 [-14.1;6.4]	not applicable
Grantham	194 [0-215]	194 [0-215]	not applicable
PolyPhen	0.999 (probably damaging)	0.999 (probably damaging)	not applicable
Align GVGD	C65 (GV:0.00 - GD:193.72)	C0 (GV:353.86 - GD:0.00)	not applicable
SIFT	score: 0, median: 4.32	score: 0, median: 4.32	not applicable
Mutation Taster	disease causing (p value: 1)	disease causing (p value: 1)	not applicable
ExAc	absent	absent	absent

Publicly available gene and protein expression data (Allen Mouse Brain Atlas and Human Brain Transcriptome Project) show particularly high levels of mGluR1 in the Purkinje cells of the cerebellar cortex, where its signaling is critically important for memory formation, motor learning, and co-ordination.^9^ Activation of mGluR1 in response to glutamatergic signaling at Purkinje cell excitatory synapses triggers a complex pathway involving inositol triphosphate receptor-dependent release of intracellular calcium. The correct function of mGluR1 in these signaling cascades is facilitated by a number of interaction partners, including the scaffold protein Homer2b.^10^ Binding of the intracellular C-terminal domain of group I mGluRs to Homer2b results in recruitment and clustering of both proteins at the plasma membrane^11^ and contributes to the organization of efficient signaling domains.^12^, ^13^ To assess the effect of the identified *GRM1* mutations on this clustering, HEK293FT cells were transiently co-transfected with mGluR1 and Homer2b followed by immunostaining. For each *GRM1* mutation, 100 cells co-expressing FLAG-tagged mGluR1 and Homer2b were counted, and the distribution of mGluR1 was classified as either “clustered” or “diffuse” based on the presence or absence of punctate staining in each cell. Representative images of this clustering are shown in Figure 2A. For further verification, immunostaining of cells using an anti-FLAG antibody was compared with immunostaining using an antibody against the N terminus of mGluR1 (Figure S1). Expression of the truncation mutation (p.Gly1056Argfs^∗^49) resulted in complete ablation of mGluR1-Homer2b clustering (Figures 2A and 2B). By contrast, the missense mutations (p.Tyr262Cys and p.Tyr792Cys) showed similar clustering patterns to wild-type (WT) mGluR1, indicating that neither of these mutations affects the clustering with Homer2b. Of note, overexpression of p.Gly1056Argfs^∗^49 mGluR1 was also associated with decreased levels of phosphorylated ERK1/2, a downstream target of activated mGluR1,^14^ when compared to WT-mGluR1 or either of the point mutations (Figure 2C).

**Figure 3:**
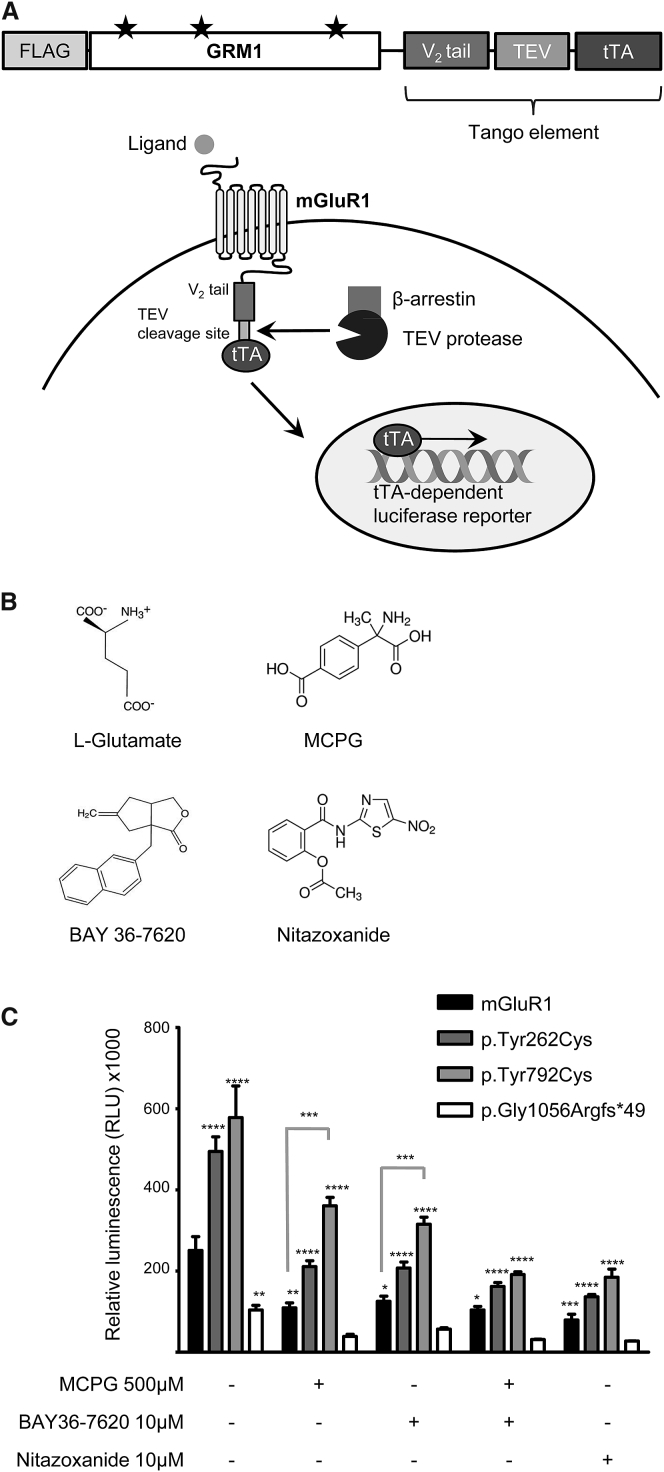
*GRM1* Mutations Affect Receptor Activity and Can Be Pharmacologically Modulated *In Vitro* (A) Overview of the modified luciferase reporter assay used to assess mGluR1 activity. The GRM1-Tango construct, into which mutations were introduced, consists of a FLAG-tagged GRM1 sequence followed by the Tango element, i.e., a V_2_ tail capable of recruiting β-arrestin, a cleavage site for the tobacco etch virus (TEV) protease, and a tetracycline-controlled transactivator (tTA). *GRM1* mutations are indicated by black stars. Signaling via mGluR1 results in a conformational change in the V_2_ tail and recruitment of β-arrestin, followed by TEV protease-mediated cleavage and release of tTA, which translocates to the nucleus and activates transcription of the luciferase reporter gene, resulting in a quantifiable output of mGluR1 activity in the form of luminescence (figure adapted from Kroeze et al.^15^). (B) Structure of the mGluR1 endogenous ligand, L-glutamate, and the inhibitors used in this study: competitive inhibitor MCPG, inverse agonist BAY36-7620, and the FDA-approved negative allosteric modulator Nitazoxanide. (C) Relative activity of mGluR1 mutants. HTLA cells, stably expressing a β-arrestin/TEV protease complex and a tTA-dependent luciferase reporter gene, were seeded at 70,000 cells/well onto poly-L-lysine-coated 96-well plates in DMEM without L-glutamine (Life Technologies), containing penicillin, streptomycin, hygromycin B, and puromycin. After 24 hr, cells were transiently transfected with the four GRM1-Tango constructs (WT, p.Tyr262Cys, p.Tyr792Cys, and p.Gly1056Argfs^∗^49) and incubated for a further 24 hr. Cells were then treated overnight with 500 μM (*RS*)-MPCG (Tocris), 10 μM BAY36-7620 (Tocris), or 10 μM Nitazoxanide (Sigma-Aldrich), diluted in assay buffer (20 mM HBSS, 1× HEPES [pH 7.4], both Life Technologies), before cell lysis in Bright-Glo solution (Promega) and luminescence reading. Data were analyzed statistically in GraphPad Prism using a two-way analysis of variance (ANOVA), followed by Bonferroni’s multiple comparison post hoc test. Significance was defined as p < 0.05 and is shown here relative to mGluR1 wild-type (WT) in the untreated condition and relative to the corresponding untreated sample for all other treatment conditions, unless otherwise indicated. Data shown are mean ± SEM from one experiment, representative of results recorded in four biological replicates, each consisting of three technical replicates per construct per condition. ^∗^p < 0.05, ^∗∗^p < 0.01, ^∗∗∗^p < 0.001, ^∗∗∗∗^p < 0.0001.

To evaluate the effects of each mutation on mGluR1 receptor activity, we employed a luciferase assay based on mGluR1-induced transcriptional activation following arrestin translocation (Tango), as previously described (Figure 3A).^15^ Consistent with the results of the immunostaining and biochemical experiments, p.Gly1056Argfs^∗^49 mGluR1 showed dramatically reduced receptor activity relative to WT (p < 0.01) (Figure 3C). Taken together, these results suggest that the truncating mGluR1 mutation causes a dominant-negative effect, resulting in a loss of receptor function and consequent disruption of downstream signaling events.

By contrast, both missense mutations resulted in significantly enhanced receptor activity compared to WT (p < 0.0001) (Figure 3C), suggesting a gain-of-function mechanism. To determine whether this effect was due to enhanced ligand sensitivity or a result of ligand-independent activation, mGluR1-transfected cells were treated with either a competitive (MCPG) or a non-competitive (BAY36-7620) antagonist of mGluR1. Treatment with either antagonist partially reduced the activity of p.Tyr262Cys and p.Tyr792Cys mGluR1 (p < 0.0001). However, a combination of both antagonists was required to reduce mutant and WT receptor activity to similar levels (Figure 3C), suggesting a role for both ligand-dependent and -independent mechanisms in the enhanced activity resulting from these mutations. These results are in keeping with the molecular genetics results and in silico pathogenicity predictions. Both missense mutations are located near regions responsible for the regulation of mGluR1 activation in response to glutamate signaling—p.Tyr262Cys in the ligand-binding domain and p.Tyr792Cys in helix VI of the transmembrane domain.^16^, ^17^ Structural analysis of mGluR1 has revealed a putative role of p.Tyr262Cys in stabilization of the open-open conformation; hence, it is possible that substitution at this position results in an increase in receptors in the active conformation.^16^ The same structural analysis has shown that helix VI undergoes a substantial conformational change during receptor activation, a process that may be disrupted by the p.Tyr792Cys substitution. As further evidence for the importance of this region, the nearby Trp798 residue has been shown to be directly involved in binding of an allosteric regulator of mGluR1 activity.^16^

Mutations that result in excessive mGluR1 signaling have been hypothesized to result in excitotoxicity via a positive-feedback mechanism, in which elevated intracellular calcium potentiates mGluR1-mediated signals.^18^, ^19^ Cerebellar Purkinje cells in particular appear acutely sensitive to these fluctuations in calcium levels, which may explain the link between gain of mGluR1 function and the development of cerebellar ataxia.^2^ Interestingly, mutations in genes encoding proteins in the mGluR1 signaling pathway, that result in activation of this pathway, have been linked to ataxia in several cases, including the *Moonwalker* ataxic mouse model^20^ and the late-onset, dominantly inherited human diseases SCA1 (MIM: 164400),^21^ SCA2 (MIM: 183090),^22^ SCA28 (MIM: 610246),^23^ and SCA41 (MIM: 616410),^24^ which share common clinical features with the individuals carrying *GRM1* missense mutations in the present study.

The *de novo* p.Gly1056Argfs^∗^49 variant, on the other hand, produces a truncated form of mGluR1 lacking the C-terminal domain. This region of the protein, containing the crucial Homer binding motif, plays an important role in receptor targeting and the regulation of signal transduction.^25^ Hence, it seems likely that deletion of the Homer binding domain would result in a loss of receptor function, and given the requirement for dimerization of the mGluR1 receptor,^26^ non-functional monomers likely exert a dominant-negative effect, accounting for the manifestation of disease in the heterozygous state.

The consequences of a loss of mGluR1 function are emphasized by results from knockout models, which show a range of developmental and functional deficits, both in the cerebellum (impaired long-term depression, movement ataxia, abnormal innervations of Purkinje cells, and compensatory mGluR5-mediated excitotoxicity)^27^, ^28^, ^29^ and in other brain regions, including impaired long-term potentiation in the hippocampus and disruption of pre-pulse inhibition.^30^, ^31^ This is further supported by recent reports of altered mGluR1 levels during neurodevelopment, in the hippocampus of a rat model of schizophrenia, highlighting the critical role of glutamate receptors across several brain regions.^32^ In humans too, mutations resulting in a loss of glutamate signaling are typically associated with a more severe neurodevelopmental phenotype. For example, individuals carrying the likely loss-of-function mutations in *GRM1* reported by Guergueltcheva et al.^5^ and Davarniya et al.^6^ or null mutations in the ionotropic glutamate receptor gene *GRID2* (MIM: 602368)^33^, ^34^, ^35^, ^36^, ^37^ all showed evidence of developmental delay and intellectual deficit in addition to cerebellar ataxia, similar to the individual carrying a *de novo GRM1* nonsense frameshift mutation described here, albeit to a larger extent. Moreover, epilepsy was described in one of the reported families with recessive inheritance^5^ and spasticity was described in one individual^5^ as well as in a spontaneous recessive *Grm1* mouse mutant.^38^ Interestingly, spasticity was also observed in one individual (family 1, III:1) reported here. Together, these phenotypes are suggestive of cortical dysfunction and point toward a critical role for mGluR1 in the development and function of additional brain regions beyond the cerebellum.

Pharmacological modulation of mGluR1 activity is attracting increasing attention as a promising therapeutic approach for the treatment of cerebellar ataxia.^3^ Indeed, negative modulators of mGluR1 activity have already been used with some success in the treatment of ataxia symptoms in mouse models.^21^, ^39^ In an attempt to identify a readily available potential therapeutic compound, we selected Nitazoxanide, an FDA-approved drug, that was identified in a recent *in silico*-*in vivo* repositioning study as a negative allosteric modulator of mGluR1/5^40^ and examined its ability to rescue the excessive mGluR1 signaling caused by the p.Tyr262Cys and p.Tyr792Cys missense variants *in vitro*. Treatment with a single 10 μM dose of Nitazoxanide proved to be a potent inhibitor of both of these mutant forms of mGluR1 in transiently transfected HEK293FT cells, as assessed by the Tango luciferase assay (Figure 3C). Given the structural similarity of its active metabolite tizoxanide to the inverse agonist BAY36-7620,^40^ it is likely that Nitazoxanide functions in a similar manner, decreasing the maximal effect of glutamate on mGluR1, regardless of the mechanism of action of the mutations.^41^ As treatment with Nitazoxanide also results in inhibition of WT receptor activity, however, *in vivo* drug titration will be required to assess therapeutic efficacy. Nonetheless, these results suggest a viable therapeutic strategy using mGlur1 inhibitors for individuals with gain-of-function mutations in *GRM1.*

In summary, we report that dominant mutations in *GRM1* cause spinocerebellar ataxia type 44 (SCA44). Our study not only emphasizes the central role of mGluR1-mediated signaling in cerebellar function, but also provides valuable insights into genotype-phenotype correlations beyond ataxia. The finding that drugs modulate mGluR1-mediated signaling in the presence of human mutations warrants further exploration of possible therapeutic avenues involving mGluR1 pathways.

## Accession Numbers

The accession number for the spinocerebellar ataxia type 44 (SCA44) reported in this paper is MIM: 617635.

## Web Resources

1000 Genomes, http://www.internationalgenome.org/
Allen Mouse Brain Atlas, http://mouse.brain-map.org
Complete Genomics, http://www.completegenomics.com
ExAC Browser, http://exac.broadinstitute.org/
HBT – Human Brain Transcriptome, http://hbatlas.org/
NHLBI Exome Sequencing Project (ESP) Exome Variant Server, http://evs.gs.washington.edu/EVS/
OMIM, http://www.omim.org/

## Supplemental Data

Document S1. Figure S1 and Supplemental Note

Table S1. Horizontal Sequence Coverage of 92 Ataxia-Associated Genes

Document S2. Article plus Supplemental Data
