# Dominant Mutations in *GRM1* Cause Spinocerebellar Ataxia Type 44

**DOI:** 10.1016/j.ajhg.2017.09.006

**Published:** 2017-10-05

**Authors:** Lauren M. Watson, Elizabeth Bamber, Ricardo Parolin Schnekenberg, Jonathan Williams, Conceição Bettencourt, Sandeep Jayawant, Jennifer Lickiss, Katherine Fawcett, Samuel Clokie, Yvonne Wallis, Penny Clouston, David Sims, Henry Houlden, Esther B.E. Becker, Andrea H. Németh

(The American Journal of Human Genetics *101*, 451–458; September 7, 2017)

Through an unfortunate oversight, Sandeep Jayawant was omitted from the author list. He is affiliated with the Department of Paediatrics, Oxford University Hospitals NHS Trust, Oxford OX3 9DU, UK, and his name appears correctly in the author list above and in the online version of the article. The authors regret this error.

In addition, the MIM accession number for SCA44 was incorrect. T the correct number is MIM: 617691, and the online version of the article has been corrected. We regret this error.

