# Dominant Mutations in *GRM1* Cause Spinocerebellar Ataxia Type 44

**DOI:** 10.1016/j.ajhg.2017.10.008

**Published:** 2017-11-02

**Authors:** Lauren M. Watson, Elizabeth Bamber, Ricardo Parolin Schnekenberg, Jonathan Williams, Conceição Bettencourt, Jennifer Lickiss, Sandeep Jayawant, Katherine Fawcett, Samuel Clokie, Yvonne Wallis, Penny Clouston, David Sims, Henry Houlden, Esther B.E. Becker, Andrea H. Németh

(The American Journal of Human Genetics *101*, 451–458; September 7, 2017)

In Figure 1C, the substitution “p.Tyr792Cys” incorrectly appeared as “p.Tyr292Cys.” This error has been corrected online. The authors regret this mistake.

