# Supplementary material for: Dominant Mutations in GRM1 Cause Spinocerebellar Ataxia Type 44
Source: Am J Hum Genet. 2017 Sep 7;101(3):451–8. doi: 10.1016/j.ajhg.2017.08.005 (PMC5591020; doi:10.1016/j.ajhg.2017.08.005)
Supplement: Document S1. Figure S1 and Supplemental Note [file mmc1.pdf]

The American Journal of Human Genetics, Volume *101*

Supplemental Data

Dominant Mutations in *GRM1* Cause Spinocerebellar Ataxia Type 44

Lauren M. Watson, Elizabeth Bamber, Ricardo Parolin Schnekenberg, Jonathan Williams, Conceição Bettencourt, Jennifer Lickiss, Katherine Fawcett, Samuel Clokie, Yvonne Wallis, Penny Clouston, David Sims, Henry Houlden, Esther B.E. Becker, and Andrea H. Németh

Supplemental Note: Case Reports

Family 1

Family 1 is a three generation English family comprising an affected mother (I:1), her son (II:1) and grand-daughter (III:1). Age of onset in the mother and son was in their 50s. The son was first seen at age 52 and complained of dysarthria, episodes of tinnitus and rotatory vertigo. He also complained of hearing loss. On examination there was no nystagmus, normal reflexes, gait ataxia and obvious dysmetria and dysdiadochokinesia. His most recent SARA score at age 61 was 9.5/40. He had no other neurological symptoms or signs, and in particular there is no evidence of cognitive involvement, and he is still working as an electronic engineer for a large car manufacturer. His mother lives in a nursing home and has not been examined, but was diagnosed with cerebellar ataxia by her local neurologist at the age of 50 after a series of falls. She also has tinnitus and hearing difficulties. Hearing loss appears to be segregating as an independent trait as there are other affected family members who have hearing loss without ataxia. MRI brain in the son revealed obvious cerebellar atrophy affecting both the hemispheres and vermis with flattening of the pons (Figure 1A, top left). The grand-daughter's onset was earlier, with complaints of balance disturbance and slow handwriting in her mid 20s. In addition she also complained of tinnitus, hearing loss and occasional migraines. On examination, she had a spastic ataxia, with a narrow-based gait and stiffness, limb ataxia and brisk reflexes with flexor plantars. There was no sensory abnormality. Her most recent SARA score at age 36 was 9/40. Her MRI brain also revealed marked cerebellar atrophy with flattening of the pons (Figure 1A, top right). Genetic testing for SCAs 1, 2, 3, 6, 7, and 17 in both son and grand-daughter did not detect any mutations.

Family 2

The proband is a male (II:1) with age at onset in his 50s of difficulties with balance and gait. He experienced a gradual progression of the disease, but twenty years into the disease he remains extremely active, exercising regularly. He generally mobilises without aids but will use two sticks if walking long distances. He has occasional falls (approximately 2-3/year). He reports occasional dysphagia with solid food and is under regular follow-up by a speech and language therapist. He does not report problems with memory or cognition. There is no sensory deficit or muscle weakness. On examination there were jerky eye movements with hypermetric saccades. Finger nose coordination was impaired bilaterally and there was bilateral dysdiadochokinesis. He had a broad-based ataxic gait. Power, tones, reflexes and sensation were normal throughout. His mother (I:2) had balance problems in her 80s with a similar slowly progressive ataxia. His sister (II:2) is similarly affected with onset of symptoms in her 50s with ataxia, and one of her daughters (III:1) is affected with age of onset in her mid 30s; no further information is currently available. MRI brain of the proband revealed subtle cerebellar atrophy (Figure 1A, bottom left). Genetic testing for SCAs 1, 2, 3, 6, 7, 12, and 17 did not detect any mutations.

Family 3

The proband (II:1) is a 5-year-old girl born to unaffected non-consanguineous parents from the UK. The child was born following normal pregnancy and delivery, but noted to have roving eye movements and delayed motor milestones shortly after birth. Walking was late (at 21 months) and was ataxic with significant falls. There is also speech and language delay. Formal IQ testing has not been performed but a formal statement of educational needs indicates mild-moderate learning difficulties. Growth parameters were in the low normal (~9th centile) range. MRI brain was normal (Figure 1A, bottom right).

Supplemental Figure

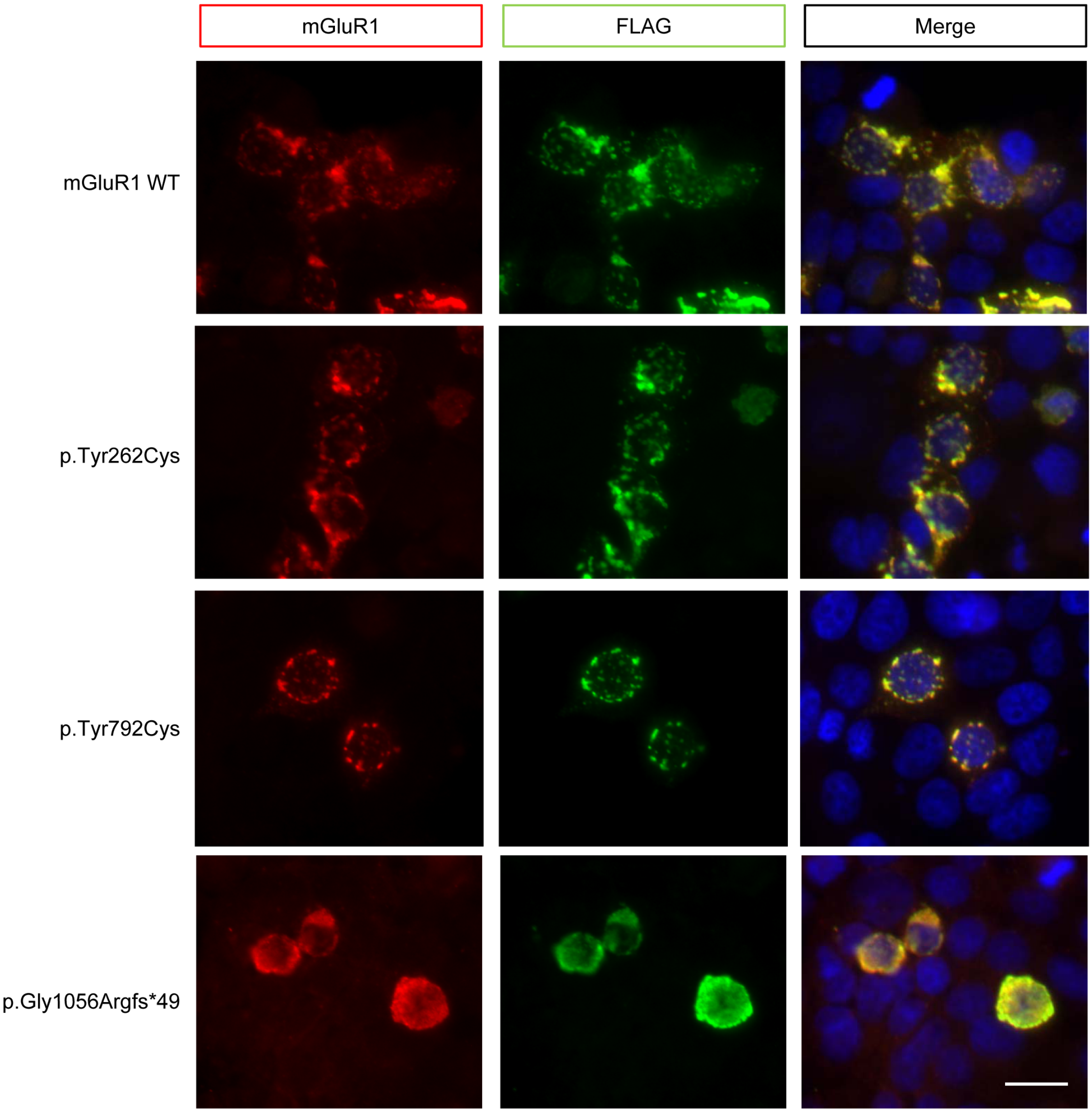

Figure S1. Immunostaining to confirm specificity of antibodies. Representative images of HEK293FT cells co-transfected with both FLAG-mGluR1 and Homer2b-MYC. Cells were fixed and co-stained with antibodies against FLAG-tagged mGluR1 (green), and the N-terminus of human mGluR1 (red). Nuclei were stained with DAPI (blue). Overlapping staining confirms the presence of mGluR1 in the membrane-bound clusters observed in Figure 2, as well as the specificity of FLAG staining. Scale bar: 20μm.
